# Supplementary material for: Patterns of Intron Gain and Loss in Fungi
Source: PLoS Biol. 2004 Nov 30;2(12):e422. doi: 10.1371/journal.pbio.0020422 (PMC532390; doi:10.1371/journal.pbio.0020422)
Supplement: Table S1 — Also available at http://genes.mit.edu/NielsenEtAl/. (4.3 MB ZIP). [file pbio.0020422.st001.zip › NielsenEtAl/html/144.html]

AN3669.1.NCU07071.1.MG00632.1.FG09275.1


```
 CLUSTAL W (1.82) Multiple Sequence Alignments - Introns Inserted


Sequence 1: NCU07071.1	356 aa
Sequence 2: MG00632.1	364 aa
Sequence 3: FG09275.1	360 aa
Sequence 4: AN3669.1	396 aa
Alignment Length: 399 aa
Number Identitical Residues: 262 aa
Alignment Score (without introns) 10408


MG00632.1 	--MMQNPYLYAHHQQHTPQGDSAWMHQQT-----THHQHAQQ-------------AAAAA
NCU07071.1	--MMQNPHVYGHHQY--PQADSAWLHQQT-----SHHQHAQ---------------AAAA
FG09275.1 	--MMPAAHVYGHHQFNPQGGDSAWMHQQPG----AHHAHTQQQQQ--------QVAAAAA
AN3669.1  	MLQTQSQHVFSHQHQY-PQADAAWLQHQQQQHQAQHHPHQAQQHSSLVAQQHAQVQAAAA
          	       :::.*::     .*:**:::*  . .: ** *  .. .:  :.. :.  ****

MG00632.1 	AASVAQQHHYSRIASAHSAANAAGLVSAAHAVGQ--------------EGGMDAGVSEDN
NCU07071.1	AANVAQQQHYNRLAGAHNNVNAL----AAATHAQ--------------DNAIEPVVSDDN
FG09275.1 	AAVQQQQQHFNRLAGAG----------QMQGHGQ--------------DADHN-NVSEDN
AN3669.1  	AAAAAQQQHYSRIAMAGTGAMTNNAAASNTTQGLGVGGVGGDGALSSAMSMIDGGVSDEN
          	**   **:*:.*:* * .   : .  :     . . .. ....: :::    :  **::*

MG00632.1 	RRTLAYIADLLNENTREAALLELSKKREQVPELALILWHSFG1VMTSLLQEIISVYTLLN
NCU07071.1	RRTLQYIADLLNENTREAALLELSKKREQVPELALILWHSFG1VMTSLLQEIISVYTLLN
FG09275.1 	RRTMAFIADLLNENTREAALLELSKKREQVPELALILWHSFG1VMTSLLQEIISVYTLLN
AN3669.1  	RKVFIWVAELLDSNRREAALMELSKKREQVPELALVIWHSFG1VMTALLQEIISVYPLLN
          	*:.: ::*:**:.* *****:**************::***** ***:*********.***

MG00632.1 	PSQLTAAASNRVCNALALLQCVASHNETRTLFLNA1HIPLYLYPFLNTTSKSRPFEYLRL
NCU07071.1	PSQLTAAASNRVCNALALLQCVASHNETRTLFLNA1HIPLFLYPFLNTTSKSRPFEYLRL
FG09275.1 	PSQLTAAASNRVCNALALLQCVASHNDTRTLFLNA1HIPLFLYPFLNTTSKSRPFEYLRL
AN3669.1  	PSQLTAAASNRVCNALALLQCVASHNETRTLFLNA1HIPLFLYPFLNTTSKSRPFEYLRL
          	**************************:******** ****:*******************

MG00632.1 	TSLGVIGALVKNDSSEVINFLLTTEIIPLCLRIMETGSELSKTVAIFIVQKILLDDNGLN
NCU07071.1	TSLGVIGALVKNDSTEVINFLLTTEIIPLCLRIMETGSELSKTVAIFIVQKILLDDNGLN
FG09275.1 	TSLGVIGALVKNDSSEVINFLLTTEIIPLCLRIMETGSELSKTVAIFIVQKILLDDNGLN
AN3669.1  	TSLGVIGALVKNDSSDVINFLLTTEIIPLCLRIMETGSELSKTVAIFIVQKILLDDIGLA
          	**************::**************************************** ** 

MG00632.1 	YICATYERFYAVGTVLSNMVAQLVEQQTARLLKHVVRCFLR2LSDNARAREALRQCLPDP
NCU07071.1	YICATYERFYAVGTVLSNMVAQLVEQQTARLLKHVVRCFLR2LSDNARAREALRQCLPEP
FG09275.1 	YICATYERFYAVGTVLSNMVAQLVESQTARLLKHVVRCFLR2LSDNARAREALRQCLPEP
AN3669.1  	YICATYERFYAVGTVLSNMVTQLVEQQTVRLLKHVVRCFLR2LSDNSRAREALRQCLPEP
          	********************:****.**.************ ****:***********:*

MG00632.1 	LRDATFSSVLRDDAATKRCLTQLLINLADNGGEP-GTGHHGL
NCU07071.1	LRDATFSSVLRDDAATKRCLAQLLINLSDNVVEP-STSGLGI
FG09275.1 	LRDATFSSVLRDDAATKRCLAQLLINLSDNVVDPNGVGGSNM
AN3669.1  	LRDATFSSVLRDDAATKRCLAQLLINLSDNVSDG--AAGVAM
          	********************:******:**  :   ..   :
```
